# Optimization of Non-Thermal Technology in Food Processing

**DOI:** 10.3390/foods15020283

**Published:** 2026-01-13

**Authors:** Milan Houška, Roman Buckow

**Affiliations:** 1 Department of Food Science, Czech Agro-Food Centre (CARC), Radiová Street 1285/7, 102 00 Prague, Czech Republic; 2 La Trobe Institute for Sustainable Agriculture and Foods, La Trobe University, Bundoora, VIC 3086, Australia

## 1. Brief Overview of Recent Developments in the Field

Any new food processing technology must first be subjected to extensive research to verify the safety and quality of the food that it creates. Following this, the effects of the various physical parameters of the technology (for example, temperature, pressure, pH, water activity) are able to be examined. When enough publications are made focusing on a particular technology, authors are able to write reviews. High-pressure processing technology [1,2,3] and pulsed electric fields [4] have gone through this process. Additionally, Reference [4] has provided a comprehensive overview of a number of modern non-thermal technologies [5].

Our Special Issue is dedicated to non-thermal food processing, but it covers only a very small part of this area of food production. These methods have a promising future as consumers are increasingly coming to prefer gentler processing methods to safeguard their health, despite that fact that these products have shorter shelf lives.

Dalla Rosa et al. [C11] provided a review for our Special Issue, considering the impact of environmentally friendly technologies on the bioavailability of various food ingredients and their safety. This review paper also focused on the overall energy consumption for processing, emphasizing that non-thermal pretreatments, such as cold plasma, pulsed electric fields, high-pressure processing and ultrasound processing, combined with mild thermal drying technology, would help reduce energy costs. Any new technology needs some energy input for their application. This has been comprehensively reviewed in Reference [6]. This paper considers the advantages of non-thermal food processing in terms of quality and carbon footprint reduction; see Table 1.

Dynamic and hydrostatic high pressure, vacuum impregnation, ultrasound, pulsed electric fields and cold plasma applications can result in a less negative effect with respect to traditional thermal treatments. These technologies can help extract important substances from secondary raw materials or waste. This ability helps the circular bioeconomy.

**Figure 1:**
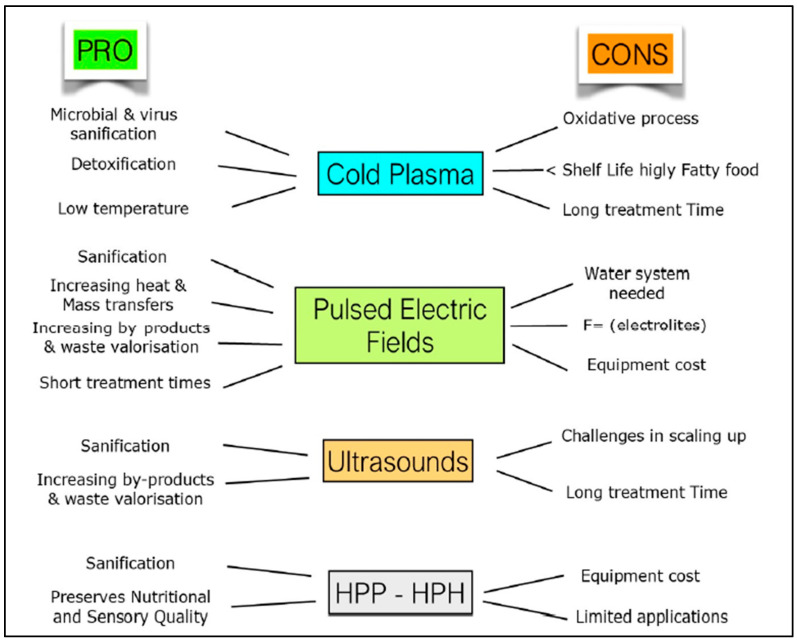
Comparison of pros and cons of investigated technologies (adapted from C11).

In Figure 1, a comparison of the positive and negative properties of the technologies considered is reported.

These treatments can produce structural changes that improve the bioaccessibility and/or the bioavailability of bioactive compounds, such as probiotic microorganisms, to improve food healthiness and the gut microbiome.

Non-thermal low-impact processing technologies could help the food industry to pursue more sustainable practices and offer better food functionality, thereby being seen more favorably in the context of the ultra-processing debate.

However, an interdisciplinary approach among food engineers, microbiologists, food chemists, bio-NMR specialists and nutritionists is necessary to discover the best processing conditions, and to increase and assess the sustainability of processing in the food industry.

Our Special Issue includes works that deal with the effects of high pressure [C1], [C4], [C5] and [C10]; high voltage pulses [C7], [C8] and [C9]; UV-C radiation [C3]; and plasma treatment [C6] and nanoparticle-based TiO_2_ [C2] on various types of food and raw materials.

We consider that the most methodologically significant publication on the verification of any new technology against classical pasteurization is Paper [7]. From a methodological point of view, it does not matter that this work is focused on the treatment of human milk. A very useful overview of new non-thermal food processing technologies is provided in Paper [6].

## 2. The Gap in Knowledge and How This Special Issue Has Addressed Those Gaps

Our Special Issue is only a small contribution to the already established knowledge of non-thermal food processing technologies. Therefore, it is very difficult to assess whether there are any gaps in the knowledge of the field on the whole. Each research piece usually reveals new problems to be solved before the studied technology can be introduced into industrial practice (see, for example, the issue of scaling up).

It can be stated that the best application in food practice has so far been achieved by high-pressure treatment technology. The most frequently used products on the market that use this technology are fruit and vegetable juices, in which important significant substances (for example, vitamins and dyes) are able to be preserved. A certain uniqueness is represented by Article [C1], which presents the treatment of human breast milk by this technology.

## 3. Primary Focus on Future Research That Should Be Considered

The future of non-thermal food processing technologies lies in researching methods that will reliably ensure the safety of products and the preservation of their most nutritionally important and healthy ingredients.

Each method must enable the implementation of a reliable system of hazard analysis and critical control points that will allow any health risks to be significantly reduced or even eliminated.

The costs of their applications in practice must be comparable to the real sales price in order to ensure commercial success.

## 4. Concluding Remarks

Gentle technologies without targeted heating can enable the food industry to maintain consumer interest in foods that maintain a high content of their original substances, such as vitamins and enzymes.

The application of these technologies requires safeguarding health and ensuring quality within the framework of the HACCP system. It is important to maintain an interdisciplinary approach between food engineers, microbiologists, chemists, machine design specialists, nutritionists and employees of regulatory authorities—all of whom work together to define standards and regulations.

## Figures and Tables

**Table 1:** Effects on food quality and carbon footprint elements of non-thermal processing (adapted from [6]).

Effects on Food Quality	Carbon Foodprint Reduction
Minimal quality loss	Less wastewater
Increasing bioavailability	Increase energy and water savings
Reduction in processing contaminants	Lower environmental impact
Maintenance of nutritional values	Decreased operational costs
Maintenance of sensorial properties	Decreased electricity
Inactivation of microorganisms	Less time-consuming
Improvement of heat and mass transfer	Inexpensive
Improvement of firmness and texture	Non-hazardous
Decreased color change	Minimal source demands
Increased shelf-life	Simple processing design
